# Laparoscopic and open complete mesocolic excision with central vascular ligation for right colonic adenocarcinoma: a retrospective comparative study

**DOI:** 10.1111/ans.17264

**Published:** 2021-10-12

**Authors:** Domenica Carmen Testa, Lorenzo Mazzola, Giuseppe di Martino, Roberto Cotellese, Federico Selvaggi

**Affiliations:** ^1^ Department of Medical, Oral and Biotechnological Sciences "G. d'Annunzio" University Chieti Italy; ^2^ Unit of General Surgery “Renzetti” Hospital Lanciano Italy; ^3^ Department of Medicine and Aging Sciences “G. d'Annunzio” University Chieti Italy; ^4^ Fondazione Villa Serena per la Ricerca Pescara Italy

**Keywords:** central vascular ligation, complete mesocolic excision, laparoscopy, minimally invasive surgery, right colon adenocarcinoma

## Abstract

**Background:**

To examine the outcome of patients treated with complete mesocolic excision (CME) with central vascular ligation (CVL) after conventional and laparoscopic surgery.

**Methods:**

We retrospectively evaluated stage I–IV colon adenocarcinoma patients treated by the same surgeon (L.M.) from 2013 to 2018. Postoperative complications, recurrences and survival are assessed.

**Results:**

Fifty‐one patients (M/F: 24/27) underwent laparoscopic right hemicolectomy with CME (L‐CME) or open CME (O‐CME) plus CVL. Tumour location was the caecum in 39.2% of cases, the transverse in 23.5%, the hepatic colonic flexure in 21.5%, and the ascending colon in 15.6%. Twenty‐four patients underwent L‐CME while 27 underwent O‐CME. More than 15 harvested lymphnodes are reported in 74.1% of O‐CME patients and in 66.7% of L‐CME patients (*p* = 0.562). Postoperative complications occurred in 7 O‐CME and 5 L‐CME patients, respectively (*p* = 0.669). Three‐year overall survival, including stage IV, was of 75% versus 77.8% for L‐CME and O‐CME patients, respectively, while for stage I–III, was of 88.9% vs. 80% in L‐CME and O‐CME, respectively (*p* = 0.440). The median follow‐up was of 2.43 years.

**Conclusion:**

CME with CVL is a meticulous, complex but feasible technique. In our experience, oncological results in terms of recurrences and overall survival, after conventional and laparoscopic CME plus CVL, are comparable. Patients with stage I–III colon adenocarcinoma have a better prognostic trend especially when more than 15 lymphnodes are removed. The respect of oncological radicality and the correct indication to minimally invasive surgery are the undiscussed key outcome variables.

## Graphical abstract

The focus is the importance of complete mesocolon excision (CME) plus central vascular ligation (CVL) during right hemicolectomy procedures. CME plus CVL is a complex operation but the implementation of this procedure in routine practice should is highly desirable.

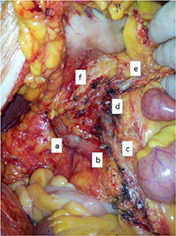

## Introduction

Colon cancer (CC) is a major public health problem, the second cause of cancer death and the third most frequent malignancy worldwide.^1^, ^2^ In Italy, CC is the second most common tumour with an incidence of right‐sided CC that accounts for 40–50% of CC occurrence.^3^, ^4^, ^5^ CME plus CVL follows established oncological principles: intact resection of the mesenterium, respect of primitive embryological layer and central lymphadenectomy.^6^ In other terms, CME plus CVL is an ‘en‐bloc’ removal of primary tumour with adequate resection margins including areas of lymphatic drainage within an intact envelope of peritoneum.^7^ CME improves oncological outcome.^6^, ^8^, ^9^, ^10^ Although the initial Italian experience documented a poor prognosis after curative right hemicolectomy for CC adenocarcinomas with 5‐year survival rate of 57%, recent results show that laparoscopic right hemicolectomy might be performed safely with a better prognosis and a 5‐year survival rate of 75%.^11^, ^12^ First described by Hohenberger in 2009, CME plus CVL surgery removes more tissue compared with standard surgery in terms of the distance between the tumour and the vascular tie, the length of large bowel and the area of mesentery.^7^, ^8^, ^13^, ^14^ Modern evidences suggest a survival benefit of 7–15% when right colectomy with CME plus CVL is performed.^7^, ^15^ The Chinese experience reported 3‐year disease‐free survival and overall survival after L‐CME for right CC of 81.7% and 89.1%, respectively.^16^ In addition, the Korean results documented 5‐year overall survival rate of 83.7% after O‐CME right hemicolectomy and 94.7% after laparoscopic resection.^17^ Currently, a survival rate of 83% is documented after 600 right total mesocolectomies in an Italian series.^18^ Variables in outcome of CC patients are the surgeon as a technician, and the pathologist as the expert of examination methods.^19^ According to this, we standardized the surgical approach by choosing a single‐surgeon's experience as first operator. The purpose of this analysis is to examine the outcome of patients treated with CME plus CVL after standardized open or laparoscopic techniques.

## Methods

**Fig. 1:**
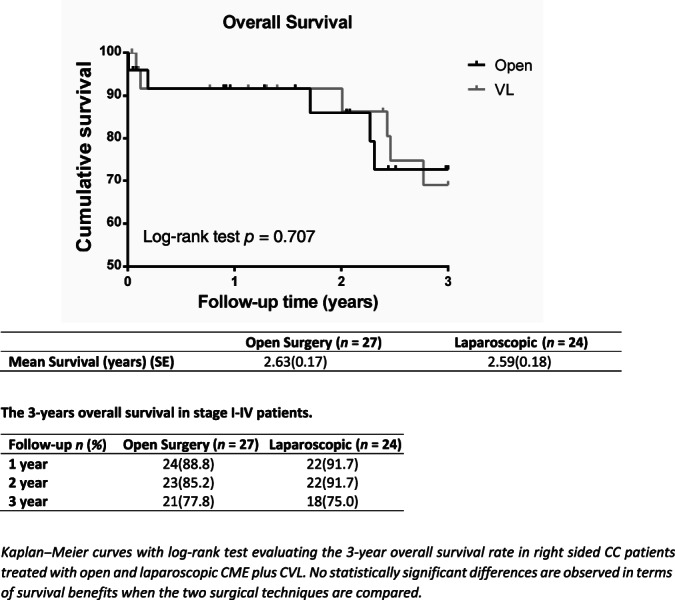
Overall survival in stage I–IV patients treated with open and laparoscopic CME plus CVL.

From February 2013 to February 2018, the same surgeon (L.M.) operated 51 patients who underwent O‐CME plus CVL and L‐CME plus CVL surgery according to their performance status, emergency conditions and after preanesthetic assessment. Laparotomy is indicated in emergency setting, in patients with previous major operation or complex abdominal wall defect and in case of multiorgan resection. Patient's characteristics such as hospital admission, length of hospital stay, postoperative complications, number of harvested lymphnodes, tumour grading and stage, local recurrence and survival rate have been analysed. A medial to lateral approach is routinely adopted. The salient surgical steps were the identification of the Treves arcade with the isolation of ileocolic vessels, the transection of the ileocolic and right colic vessels, and the ‘en‐bloc’ lymphadenectomy from ileocolic vessels to gastrocolic trunk of Henle. Total right mesocolectomy is performed in cases of caecal or ascending CC with ligation of the right branch of the middle colic vessels (Fig. 1). In addition, the extended right colectomy is performed for hepatic flexure or proximal transverse CC. In both surgical procedures, the ileum is stapled at 10–15 cm from the ileocaecal valve and the surgical specimen is extracted through a protected 3–5 cm periumbilical incision. In all cases, side‐to‐side, isoperistaltic manual extra‐corporeal anastomosis (ECA) in double layer suture is performed. Quantitative variables are summarized as mean and standard deviation (SD) or median and interquartile range (IQR), according to their distribution. In addition, categorical variables are summarized as frequency and percentage. Student's t‐test or Mann–Whitney *U* test are used to compare continuous variables between studied groups as appropriate. Categorical variables are compared using Pearson's Chi‐Square test. Spearman's correlation coefficient is calculated to assess correlation among continuous variables. Kaplan–Meyer methods with log‐rank test is performed to evaluate overall survival between both groups. All tests are considered statistically significant for a *p*‐value less than 0.05. All analyses are performed with the IBM SPSS for Statistics software v23.

## Results

**Fig. 2:**
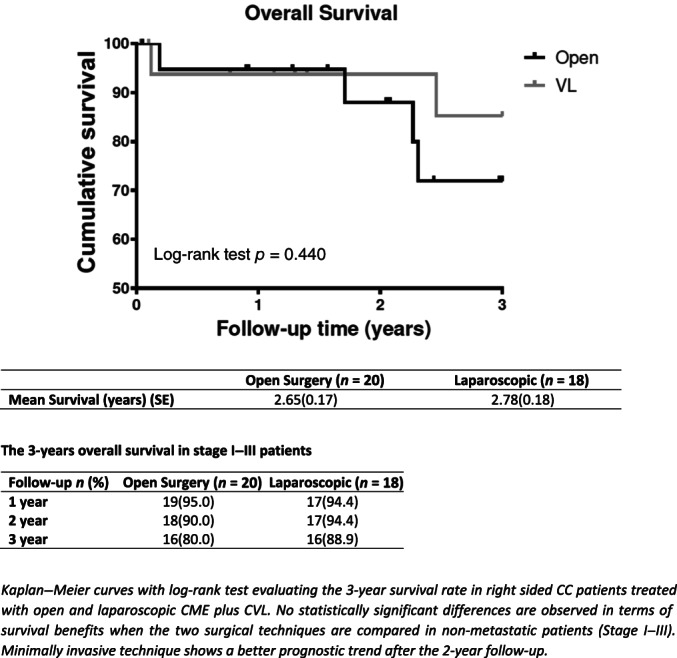
Overall survival in stage I–III patients treated with open and laparoscopic CME plus CVL.

**Fig. 3:**
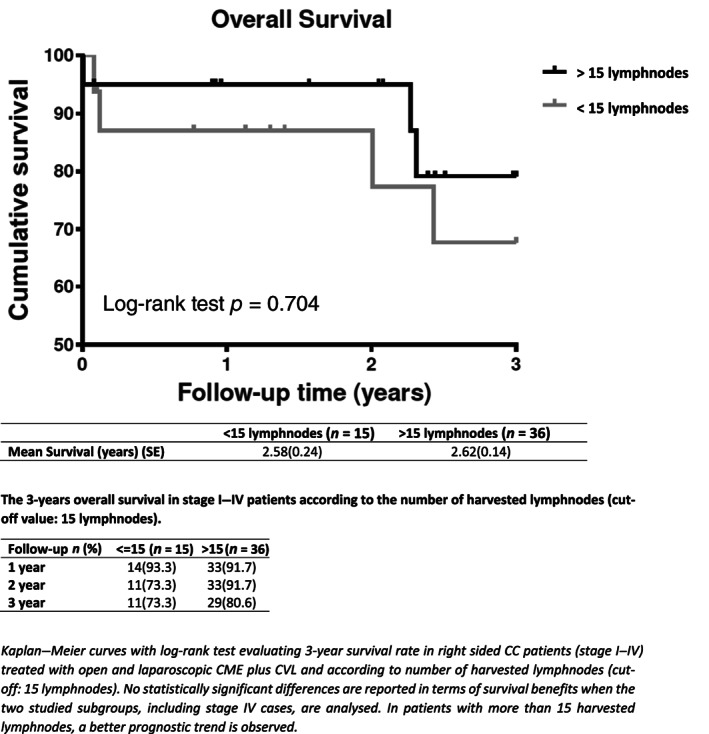
Overall survival in stage I–IV patients treated with open and laparoscopic CME plus CVL according to harvested lymphnodes.

**Fig. 4:**
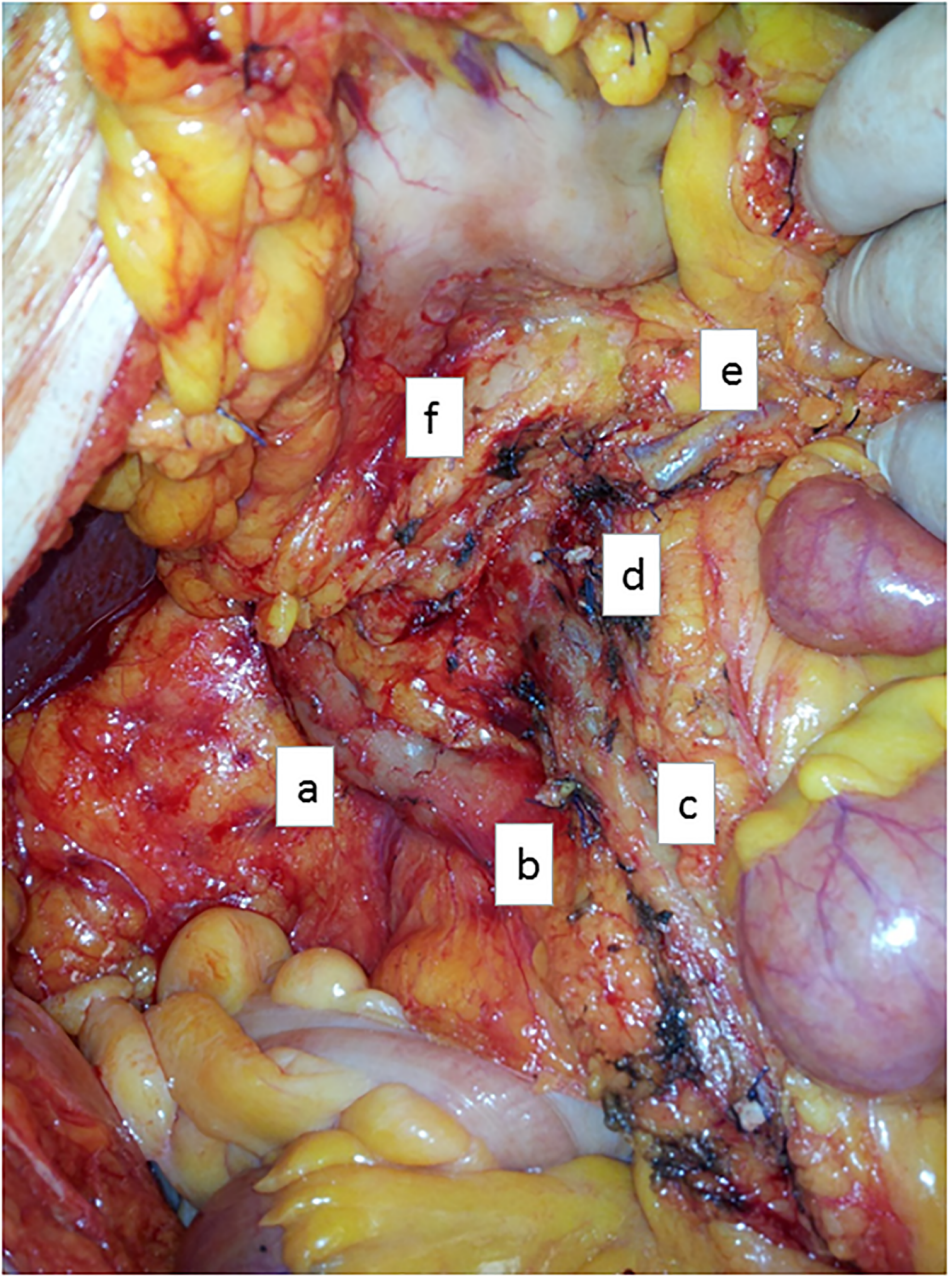
Operative field view of conventional CME surgery. (a) duodenum; (b) ileo‐colic vein; (c) superior mesenteric vein; (d) right colic vessels; (e) middle colic vein (its left branch); (f) inferior border of the pancreas.

Fifty‐one consecutive patients with right‐sided CC underwent O‐CME or L‐CME. The O‐CME plus CVL group consisted of 27 patients (11 males and 16 females) with a mean age of 73.6 ± 11.8 years, while the L‐CME plus CVL group consisted of 24 patients (11 males and 13 females) with a mean age of 76.3 ± 7.8 years (Table 1). No statistical difference in gender (*p* = 0.714) and age (*p* = 0.334) between the two groups is observed. At the admission, 15 patients presented bowel obstructive symptoms (7 in O‐CME and 8 in L‐CME), 15 patients presented lower gastrointestinal bleeding (10 in O‐CME and 5 in L‐CME), and in 21 patients atypical symptoms like abdominal pain, vomiting, and constipation are documented (10 in O‐CME vs. 11 in L‐CME, respectively). Elderly patients (age >80 years) were 37.2%. At the histology, 40.7% of O‐CME patients and 75% of L‐CME group (11 vs. 18 patients) had grade 2 tumour (moderately differentiated adenocarcinoma), while 59.3% of patients in the O‐CME group and 25% in the L‐CME group (16 vs. 6 patients) had grade 3 tumour (poorly differentiated adenocarcinomas) (Table 1). There was no statistically significant difference between the two groups in tumour stage (*p* = 0.496), with a total of 9 patients for stage I, 16 for stage II, 13 for stage III and 13 patients for stage IV. Mean operative time was significantly different between O‐CME and L‐CME patients [185 min (range: 165–210) vs. 252 minutes (range: 221–273), respectively, *p* < 0.001] (Table 2). The tumour dimension was similar in O‐CME and L‐CME patients (6 cm, IQR 4–7 cm vs. 5 cm IQR 4–6 cm, respectively, *p* = 0.247), as the number of harvested lymphnodes (median: 19 lymphnodes, interquartile range 14–24 vs. 21 lymphnodes, interquartile range 14–27, in O‐CME and L‐CME respectively, *p* = 0.664). Interestingly, 74.1% of O‐CME patients and 66.7% of L‐CME patients had more than 15 lymphnodes harvested (*p* = 0.562) (Table 2). Tumour specific location was the caecum in 39.2% of cases, the transverse in 23.5%, the right hepatic flexure in 21.5%, and the ascending colon in 15.6% (Table 3). Postoperative complications occurred in 7 and 5 patients in O‐CME and L‐CME group, respectively (*p* = 0.669), including 1 case of anastomotic leak, 1 case of anastomotic bleeding that required re‐laparotomy, 1 case of biliary fistula and 2 abdominal collections. Overall perioperative mortality was 3.9%. The hospital stay was similar (12 days vs. 8 days in the O‐CME and L‐CME patients, respectively, *p* = 0.029), with non‐significant correlation between age and hospital stay (Rho 0.231, *p* = 0.103). In addition, 37% of O‐CME patients and 41.7% of L‐CME patients are treated with adjuvant chemotherapy (*p* = 0.721). Local recurrence was 7.4% in the O‐CME and 8.3% in the L‐CME group (*p* = 0.902) (Table 1). There was no statistical difference for tumour dimension between patients with and without local recurrence (*p* = 0.545). Distant recurrence was observed in 4 (14.8%) and 4 (14.3%) patients in the O‐CME and L‐CME group, respectively (*p* = 0.856) (Table 1). After 3‐years, the overall survival was 77.8% for O‐CME *vs*. 75% for L‐CME, respectively (including stage IV) (Fig. 2). With the exclusion of stage IV, the 3‐year overall survival was 80% for O‐CME vs. 88.9% for L‐CME patients, respectively (Fig. 3). In stage I‐III patients with more than 15 harvested lymphnodes, a better prognostic trend is observed after 2‐year follow‐up, even if data did not reach statistical significance (Fig. 4). The median follow‐up was of 2.43 years (2.31 vs. 2.62 in O‐CME and L‐CME patients, respectively).

**Table 1:** Baseline characteristics

	Open surgery (*n* = 27)	Laparoscopic surgery (*n* = 24)	*p*‐value^a^
Age *m* ± *SD*	73.6 ± 11.8	76.3 ± 7.8	0.334^b^
Gender *n* (%)			0.714
M	11 (40.7)	11 (45.8)	
F	16 (59.3)	13 (54.2)	
ASA Score *n* (%)			0.707
2	10 (37.0)	7 (29.2)	
3	11 (40.7)	10 (41.7)	
4	1 (3.7)	2 (8.3)	
Missing	5 (18.5)	5 (20.8)	
Symptoms at the admission *n* (%))			0.447
Obstruction	7 (26.0)	8 (33.3)	
Bleeding	10 (37.0)	5 (20.8)	
Others	10 (37.0)	11 (45.8)	
Chemotherapy *n* (%)	10 (37.0)	10 (41.7)	0.721
Tumour grading *n* (%)			**0.014**
1	–	–	
2	11 (40.7)	18 (75.0)	
3	16 (59.3)	6 (25.0)	
Tumour staging *n* (%)			0.496
I	4 (14.8)	5 (20.8)	
IIA	8 (29.6)	5 (20.8)	
IIB	–	3(12.5)	
IIIA	3 (11.1)	1 (4.2)	
IIIB	4 (14.8)	2 (8.3)	
IIIC	1 (3.7)	2 (8.3)	
IVA	6 (22.2)	4 (16.7)	
IVB	1 (3.8)	2 (9.5)	
Distant recurrence *n* (%)	4 (14.8)	4 (14.3)	0.856
Local recurrence *n* (%) (excluding T4)	–	1 (4.2)	
Total local recurrence *n* (%) Elderly patients >80 years *n*(%)	2 (7.4) 9 (33)	2 (8.3) 10 (42)	0.902

*Note*: Data are reported as mean ± standard deviation or percentage of patients. Tumour grading: 1 well differentiated, 2 moderately differentiated, 3 poorly differentiated. Tumour staging according to AJCC colon cancer staging.

^a^ Pearson's Chi‐square test.

^b^ Student's *t*‐test.

**Table 2:** Surgical characteristics

	Open surgery (*n* = 27)	Laparoscopic surgery (*n* = 24)	*p*‐value^a^
Surgery time median (min) (IQR)	185 (165–210)	252 (221–273)	**<0.001**
Tumour dimension median (cm) (IQR)	6 (4–7)	5 (4–6)	0.247
Removed lymphnodes ≥15 (num) (%)	20 (74.1)	16 (66.7)	0.562^b^
Overall complications (num) (%)	7 (25.9)	5 (20.8)	0.669^b^
Length of stay median (day) (IQR)	12 (9–15)	8 (6–12)	0.029

	No local recurrence (*n* = 47)	Local recurrence (*n* = 4)	*p*‐value
Tumour dimension (cm) QR	5 (4–7)	7 (3–7)	0.545

*Note*: Data are reported as median and interquartile range (IQR) or percentage.

^a^ Mann–Whitney *U* test.

^b^ Pearson's Chi‐square test.

**Table 3:** Tumour site and lymphnode metastasis

	Number of patients	Number of N+ patients	%
Caecum	20	10	50%
Ascending colon	8	4	50%
Hepatic flexure	11	5	45%
Transverse colon	12	4	33.3%

*Note*: Data are reported as number of patients and percentage. N+ patients were all cases that presented pericolic positive lymphnode metastasis.

## Discussion

The concept of CME with CVL is based on the complete removal of the mesentery and the central vascular tie with all lymphnodes draining the tumour area.^14^, ^20^, ^21^, ^22^ CME provides superior specimens, acceptable morbidity and improves overall survival.^3^, ^17^, ^22^, ^23^ The main difficulty in performing CME plus CVL right hemicolectomy is to identify the gastrocolic trunk of Henle, its anatomic variations and the dissection close to the superior mesenteric vein.^24^, ^25^, ^26^ The first report on laparoscopic right colectomy appeared in 1991 and after only 1 year, laparoscopic right hemicolectomy with intracorporeal ileocolic anastomosis (ICA) was described.^27^ Recent results confirm that L‐CME is a safe and effective alternative associated with excellent oncologic outcomes and acceptable complications.^9^, ^15^, ^28^, ^29^, ^30^, ^31^ In our experience, the choice of performing O‐CME plus CVL or L‐CME plus CVL strongly depends on emergency setting and preanesthetic evaluation. In frail older patients with severe comorbidity and high risk of perioperative mortality, we preferred to perform O‐CME plus CVL. In the literature, the reported postoperative complications are around 21% in the CME patients and around 18% in non‐CME patients, respectively.^2^, ^16^ Anastomotic leakage varies from 1.1% to 5% of cases and in CME patients, it is around 1.7%.^1^, ^2^, ^16^, ^29^ In laparoscopic right hemicolectomy with ICA, the rate of anastomotic leak range from 0% to 8.6% while after ECA the rate range from 0 to 5.8%.^27^, ^32^, ^33^ The selection between laparoscopic right colectomy with ECA and totally laparoscopic procedures with ICA is still a hot topic.^34^ Although modern literature reported similar outcome after ICA and ECA during laparoscopic right hemicolectomy,^33^, ^34^ we preferred to perform ECA to reduce operative time and to standardized surgical procedure between the two groups. After right hemicolectomy, the reported superior mesenteric vein damage is around 1.6% and the conversion to O‐CME surgery is around 13%.^7^, ^26^ In our series, no cases have been converted into laparotomy and the observed complications included one case of anastomotic leak, 1 case of anastomotic bleeding that required re‐laparotomy, 1 case of biliary fistula and two cases of intra‐abdominal collections. After L‐CME, the reported total hospital stay is of 12 days with a range of 6–20 days.^16^, ^23^, ^35^ For selected patients, the L‐CME reduces the hospital stay with a mean difference of 4.07 days compared with O‐CME surgery.^28^ In our experience, O‐CME patients had a mean hospital stay of 12 days compared with 8 days of L‐CME patients as reported in Table 2. In performing laparoscopic right hemicolectomy, the reported mean operating time was of 119 ± 38 min; the mean length of resected colon was of 27.8 ± 4.48 cm, and the average width of the clear margins of 6.8 ± 5.3 cm.^12^ Similar operative time between O‐CME and L‐CME patients are reported by Kim and co‐workers (175 vs. 178 min).^26^ In our analysis, the mean operative time was significantly different between O‐CME plus CVL and L‐CME plus CVL groups [185 min (range: 165–210) vs. 252 min (range: 221–273), respectively, *p* < 0.001]. This strongly reflects our initial enthusiasm in performing minimally invasive CME surgery. Both laparoscopic right colectomy with ICA and ECA are oncologically adequate.^27^ The main potential advantages of CME with ICA seems to be in obese patients, by reducing the accidental mesenteric twists.^27^, ^36^ In our experience, any mesenteric volvulus is documented and all cases are treated with ECA in double layer suture. After CC resection, the most common sites of systemic recurrence were liver, peritoneum, para‐aortic lymphnodes, lung and ovary.^17^ In the literature, the 5.2‐year cumulative incidence of recurrence was 9.7% in CME group compared with 17.9% in non‐CME patients, and the absolute risk reduction of CME after 5.2 years was 8.2%.^37^ In our clinical practice, recurrences were common in advanced tumour staging in both groups without statistical significance (Table 1). Lymphnode metastases is reported in up to 11% of cases. Specifically, metastases in the sub‐pyloric lymphnodes is detected in 1.1–3.8% of cases, while metastasis to lymphnodes along the right branch of the middle colic artery occurs in 6.1% of patients with ceacal cancer and in approximately 10% of the patients with transverse cancer, along right colic artery.^20^, ^38^ Conventional non‐CME surgery has a morbidity of 12.1–28.5% and a 3.7% mortality risk versus 12–36.4% morbidity and 2.1–3% mortality for O‐CME.^1^ In L‐CME, the morbidity is 4–31% with a mortality of 0.5–0.9%.^1^ In our study, where the subgroup of elderly patients was more than 37%, overall perioperative mortality was 3.9% and the 3‐year overall survival was 80% in O‐CME and 88.9% in L‐CME patients with stage I‐III. Our personal experience has significant limitations such as its retrospective nature and its relative small numbers of patients, but has the peculiarity of comparing data coming from the same operating surgeon and his standardized techniques. Further prospective studies will provide a stronger validity of these considerations.

## Conflict of interest

None declared.

## Ethics statement

This study is approved by Comitato Etico delle Province di Chieti e Pescara.

## Author Conntributions

**Domenica Testa:** Data curation; investigation. **Lorenzo Mazzola:** Supervision; validation. **Giuseppe di Martino:** Data curation; formal analysis. **Roberto Cotellese:** Methodology; supervision. **Federico Selvaggi:** Conceptualization; project administration; supervision.
